# Immunomodulation and biofilm disruption of piezoelectric materials in bone infection

**DOI:** 10.3389/fcell.2026.1976300

**Published:** 2026-10-16

**Authors:** Zhen Wang, Kai Zheng, Yunfeng Tang, Zhen Zhang, Zhou Zhong, Dong Wang, Zijian Chen, Ye Wang, Zan Chen, Pengrui Feng, Wenjie Huang, Mengkun Liu, Yun Hao, Feng Yu, Guanglin Wang, Min Yi, Xiangtian Deng

**Affiliations:** 1 Trauma Medical Center, Department of Orthopedic Surgery, West China Hospital, Sichuan University, Chengdu, China; 2 Department of Orthopedics, West China Hospital, Sichuan University, Chengdu, China; 3 Operating Room, Department of Anesthesiology, West China Hospital, Sichuan University, Chengdu, China; 4 School of Nursing, Sichuan University, Chengdu, China

**Keywords:** biofilm disruption, bone infection, bone regeneration, immunomodulation, macrophage polarization, osteomyelitis, piezoelectric materials, reactive oxygen species

## Abstract

Bone infection remains a persistent clinical challenge due to biofilm formation, antibiotic resistance, and impaired bone regeneration. This manuscript focuses on the application of piezoelectric biomaterials in the treatment of bone infection and systematically elucidates their ability to convert *in vivo* mechanical stimulation into localized electrical signals and, in catalytically active systems, promote reactive oxygen species generation, thereby enabling efficient bacterial eradication and biofilm disruption. We further synthesize recent evidence showing that piezoelectric stimulation can modulate the infection-associated immune microenvironment and support osteogenesis and angiogenesis. Rather than considering antibacterial, immunomodulatory, and regenerative effects as parallel functions, we integrate them into a stage-adaptive therapeutic framework in which early biofilm disruption, bacterial eradication, and antimicrobial immune activation are followed by oxidative-stress control, inflammation resolution, pro-reparative immune remodeling, angiogenesis, and osteogenesis. This pathophysiology-oriented perspective highlights the potential of piezoelectric materials to coordinate the sequential therapeutic requirements of infected bone repair. Finally, current challenges and future perspectives regarding material design optimization, mechanistic understanding, and clinical translation are discussed.

## 1 Introduction

Osteomyelitis (OM) is an inflammatory disease characterized by infection of the bone marrow as well as the surrounding bone and soft tissues caused by pathogenic microorganisms (Massaccesi et al., 2022). The etiological factors of OM are diverse and may include infections caused by bacteria, viruses, parasites, or fungi; however, bacterial infection is the most common in clinical practice. Gram-positive staphylococci are the predominant pathogens responsible for OM, among which *Staphylococcus aureus* accounts for approximately 44%–75% of infection cases and is particularly prevalent in chronic osteomyelitis (Song et al., 2025). According to the onset and clinical course, OM is generally classified into acute, subacute, and chronic types (Pimentel de Araujo et al., 2021; Kavanagh et al., 2018). The pathological characteristics of OM are manifested as inflammation-driven imbalance of bone remodeling and destruction of bone tissue, which are closely associated with enhanced osteoclast activity and secondary osteonecrosis. Currently, the treatment of OM mainly relies on surgical debridement and antibiotic therapy; however, many patients eventually develop chronic osteomyelitis, largely due to bacterial biofilm formation and dysregulated host immune responses (Masters et al., 2019).

Biofilms are bacterial communities that adhere to implant surfaces, necrotic tissues, or other favorable microenvironments (Khatoon et al., 2018). The formation of biofilms enables bacteria to evade both innate and adaptive immune responses, thereby conferring tolerance to host defenses and antimicrobial therapies. During osteomyelitis infection, bacteria initially adhere to host tissues through multiple adhesion factors, allowing them to escape clearance by the immune system and mucosal barriers (Masters et al., 2019). In addition, bacteria can express various surface-anchored proteins to enhance resistance to host immune responses, among which *Staphylococcus aureus* protein A is one of the most representative immune evasion factors (Geoghegan and Foster, 2017). These surface-anchored proteins are extensively involved in host cell adhesion, biofilm formation, and fibrin binding, further promoting biofilm development and thereby exacerbating the persistence and refractoriness of infection.

With the emergence of novel biomaterials, encouraging therapeutic outcomes have been achieved in OM treatment. Among these emerging materials, piezoelectric materials play a pivotal role in combating bacterial infections. Piezoelectric materials are functional materials capable of converting mechanical stimulation into electrical stimulation (Chen B. et al., 2025). Their key feature lies in the non-centrosymmetric crystal lattice structure. When subjected to mechanical force, crystal deformation leads to the separation of positive and negative charge centers, generating electric dipoles. Currently, piezoelectric materials have been applied across multiple disciplines, including serving as nanogenerators for soft tissue repair (Kao et al., 2022). More importantly, piezoelectric materials have been directly investigated as antibacterial and antibiofilm platforms. Under mechanical stimulation, such as ultrasound, vibration, or endogenous biomechanical forces, piezoelectric polarization generates localized surface charges and electrical signals. In piezocatalytically active systems, the resulting charge separation can further drive redox reactions and promote the generation of reactive oxygen species (ROS). These mechanically activated electrical and catalytic effects can disrupt extracellular polymeric substances and biofilm architecture, impair bacterial membrane integrity, interfere with intracellular redox and respiratory processes, and ultimately contribute to bacterial eradication (Chen et al., 2024; Chen et al., 2023; Chen et al., 2026). Importantly, the antibacterial activity of piezoelectric systems is not necessarily attributable to ROS alone, as electrical perturbation, mechanical disruption, interfacial electron transfer, ion release, and other material-dependent effects may also contribute. Several recent reviews have summarized piezoelectric materials for anti-infective biomedical applications, including their material classifications, piezocatalytic antibacterial mechanisms, structural design strategies, and roles in tissue repair (Chen et al., 2024; Chen et al., 2023; Chen et al., 2026). However, existing discussions have largely been organized according to material type, antibacterial mechanism, or application category. The dynamic pathophysiological sequence of bone infection has received comparatively less integrated consideration. In particular, persistent biofilms, dysregulated antimicrobial immunity, excessive or unresolved inflammation, and impaired angiogenesis and osteogenesis are not independent events, but interconnected processes that evolve across different stages of infection control and bone repair. Therefore, a framework linking these sequential therapeutic requirements is needed to better understand how piezoelectric materials may be rationally designed for bone infection. Accordingly, rather than simply cataloguing piezoelectric materials and their antibacterial effects, this review examines their therapeutic roles along the pathological continuum of bone infection. We organize the available evidence around three interconnected dimensions: direct bacterial and biofilm elimination, regulation of the infection-associated immune microenvironment, and restoration of osteogenic and angiogenic regeneration. On this basis, we further propose a stage-adaptive therapeutic framework in which the early infection-control phase prioritizes biofilm disruption, bacterial eradication, and antimicrobial immune functions, whereas the subsequent repair phase emphasizes control of excessive oxidative stress, resolution of persistent inflammation, metabolic and immune reprogramming, angiogenesis, and osteogenesis. Through this framework, we aim to provide a pathophysiology-oriented perspective for the design of next-generation piezoelectric biomaterials for bone infection.

## 2 Basic properties of piezoelectric materials

Piezoelectric materials are a class of functional materials capable of converting mechanical energy into electrical energy and *vice versa*. At present, these materials have evolved from laboratory-scale physical discoveries into cornerstones of modern industry. Whether in advanced medical ultrasound equipment, precision actuators for aerospace applications, or radio-frequency filters in everyday smartphones, piezoelectric materials play an irreplaceable role. The essence of the piezoelectric effect originates from the non-centrosymmetric nature of crystal structures (Chen et al., 2024; Ali et al., 2023; Aydin and Çelebi, 2023; Li et al., 2023; Qian et al., 2020). The piezoelectric effect can be divided into the direct piezoelectric effect and the inverse piezoelectric effect (Zhu et al., 2021). The direct piezoelectric effect refers to the generation of bound surface charges when a material undergoes mechanical deformation under an external force, as the internal electric dipole moments are altered. The inverse piezoelectric effect occurs when an external electric field is applied to the material, inducing dipole displacement and consequently causing macroscopic geometric deformation of the material. From a biomedical application perspective, piezoelectric materials relevant to bone infection can be organized into three major platform groups: (1) inorganic piezoelectric materials, including lead-free piezoelectric ceramics, wurtzite piezoelectric semiconductors, and two-dimensional piezoelectric semiconductors; (2) polymeric piezoelectric materials, including non-biodegradable and biodegradable piezoelectric polymers; and (3) composite and heterojunction piezoelectric platforms that integrate multiple piezoelectric, catalytic, structural, or bioactive components. Although composite and heterojunction systems do not constitute a fundamental compositional class in the strict physicochemical sense, they are considered separately here because they represent an increasingly important biomedical platform and enable enhanced charge separation, improved mechanical compatibility, and integration of multiple therapeutic functions. A more detailed comparison of these categories is provided in Table 1.

**TABLE 1:** Representative piezoelectric material platforms and their characteristics relevant to bone infection therapy.

Material/platform category	Representative materials	Piezoelectric/functional basis	Principal advantages	Key limitations	Reported or potential roles in bone infection	Representative refs
Lead-free piezoelectric ceramics	BTO, BSTO, KNN, and phase-/defect-engineered derivatives	Non-centrosymmetric perovskite structures generate piezoelectric polarization under mechanical stimulation. Compositional regulation, phase engineering, elemental doping, and defect engineering can enhance polarization, charge separation, and piezocatalytic activity	Relatively strong and tunable piezoelectric output; good physicochemical stability and generally favorable biocompatibility; suitable for nanoparticles, coatings, and 3D-printed scaffolds	Intrinsic brittleness and limited biodegradability; long-term retention and degradation require evaluation; performance depends on crystalline phase, polarization state, grain size, and defect concentration; complex fabrication may compromise reproducibility	Ultrasound-triggered electrical stimulation and ROS production; bacterial killing and biofilm disruption; macrophage functional regulation; neuro-osteogenic coupling; multifunctional antibacterial and osteogenic scaffolds for infected bone-defect repair	(Khare et al., 2020; Persano et al., 2024; Huang et al., 2024; Yu et al., 2023; Chen K. et al., 2025; Wu et al., 2024; Zheng et al., 2025; Xu et al., 2025)
Wurtzite piezoelectric semiconductors	ZnO nanorods, nanowires, and nanoparticles	The non-centrosymmetric wurtzite structure generates piezoelectric polarization through displacement of Zn^2+^ and O^2-^ charge centers under mechanical deformation. ZnO dissolution may additionally release Zn^2+^ and provide ion-mediated biological effects	Relatively inexpensive; readily synthesized in diverse nanostructures; combines piezoelectric ROS generation with Zn^2+^-mediated antibacterial and osteogenic activity at controlled doses; readily integrated into composite scaffolds	Dissolution and excessive Zn^2+^ release may cause cytotoxicity; performance depends on morphology, crystallinity, dose, and stimulation conditions; long-term *in vivo* fate requires clarification	Direct bacterial killing, membrane damage, and biofilm inhibition; Zn^2+^-mediated antibacterial activity; enhancement of piezoelectric, antibacterial, and osteogenic performance in composite scaffolds	(Ferrone et al., 2019; He and Briscoe, 2024; Wang C et al., 2022; Chen K. et al., 2025; Zheng et al., 2025; Cheng et al., 2025)
Two-dimensional piezoelectric semiconductors	Monolayer and odd-layer MoS_2_ nanosheets	Broken inversion symmetry in monolayer and odd-layer MoS_2_ generates an in-plane piezoelectric response. Large surface area and tunable interfaces facilitate charge separation, electron transfer, and catalytic reactions	Large interfacial area; mechanical flexibility; interfacial tunability; favorable catalytic properties; suitable for piezoelectric heterojunctions and piezo-augmented sonosensitizers	Piezoelectricity depends on layer number, orientation, and structural integrity; aggregation may reduce activity; *in vivo* degradation, clearance, long-term retention, and biosafety remain insufficiently defined	Piezo-augmented sonodynamic therapy; enhanced interfacial charge separation and ROS production; increased mechanical contact with bacteria; MRSA/biofilm eradication and experimental osteomyelitis treatment	(Hoàng Ly et al., 2025; Singh et al., 2019; Wang Y. et al., 2022; Feng et al., 2022)
Non-biodegradable piezoelectric polymers	PVDF and P(VDF-TrFE)	Ordered molecular dipoles in the electroactive β phase generate piezoelectric polarization. Incorporation of TrFE favors electroactive-phase formation and improves electromechanical coupling	Flexible, lightweight, chemically stable, and readily processable; suitable for electrospinning and fabrication of fibers, membranes, coatings, sensors, and flexible composite scaffolds	Limited biodegradability may restrict long-term implantation; performance depends on β-phase content, molecular orientation, polarization, and processing conditions; intrinsic antibacterial activity is limited without additional functional components	Flexible electroactive coatings and membranes; potential reduction of implant-associated bacterial adhesion after functionalization; piezoelectric fibers and composite scaffolds for local electrical stimulation in bone repair	(Zhang M. et al., 2023; Pourmadadi et al., 2025; Mohammadpourfazeli et al., 2022)
Biodegradable piezoelectric polymers	PLLA, PDLA, PHB, and their piezoelectric composites	Non-centrosymmetric molecular organization and oriented dipoles in stereoregular or semicrystalline biodegradable polymers can generate shear piezoelectric responses. In polylactides, molecular chirality and orientation of carbonyl dipoles are particularly important, whereas piezoelectric performance generally depends on crystallinity, chain orientation, and processing conditions	Biodegradable and generally biocompatible; mechanically flexible and processable; suitable for temporary or resorbable electroactive implants and tissue-engineering scaffolds	Piezoelectric output is generally lower than that of many ceramics; performance depends on crystallinity, molecular orientation, processing history, and loading mode; degradation may alter electrical and mechanical properties over time	Resorbable electroactive fibers, membranes, and scaffolds; mechanically generated electrical stimulation; support for cell adhesion, osteogenic differentiation, and tissue regeneration; multifunctional composites may additionally provide antibacterial activity	(Dai et al., 2022; Zaszczyńska et al., 2024; Goonoo et al., 2017; Zhang Y. Q. et al., 2023; Iwuozor et al., 2025; Zhang H. et al., 2025; Lotz et al., 2018; Yoshida et al., 2014; Mirghaffari et al., 2025; Schönlein et al., 2026; Zhang S. et al., 2024; Cheng et al., 2025)
Composite and heterojunction platforms	Ceramic–polymer composites, piezoelectric heterojunctions, multifunctional scaffolds, Janus membranes, and hydrogels	Interfacial electric fields, band alignment, defect engineering, matrix integration, and multicomponent catalytic reactions enhance charge separation, ROS generation, mechanical compatibility, and biological functionality	Integrate antibacterial, antibiofilm, immunomodulatory, drug-delivery, antioxidant, angiogenic, and osteogenic functions within one platform; enable spatially, temporally, or intensity-dependent therapeutic responses	Multicomponent fabrication increases structural complexity; batch-to-batch reproducibility, sterilization stability, component degradation, dose control, scale-up, and regulatory assessment remain challenging; individual component contributions may be difficult to distinguish	ROS-mediated bacterial killing and biofilm disruption; on-demand local drug release; immune-microenvironment remodeling; antioxidant protection; angiogenesis, osteogenesis, and vascularized bone regeneration	(Huang et al., 2024; Wang et al., 2025; Feng et al., 2022; Yu et al., 2023; Chen B. et al., 2025; Zhang G. et al., 2025; Roy et al., 2025; Xi et al., 2025; Pan et al., 2026; Chen K. et al., 2025; Wu et al., 2024; Zheng et al., 2025; Xu et al., 2025; Fan et al., 2025; Cheng et al., 2025)

Abbreviations: BTO, barium titanate; BSTO, barium strontium titanate; KNN, potassium sodium niobate; MRSA, methicillin-resistant *Staphylococcus aureus*; PLLA, poly-L-lactic acid; PVDF, polyvinylidene fluoride; P(VDF-TrFE), poly (vinylidene fluoride–trifluoroethylene); ROS, reactive oxygen species.

The categories used here are application-oriented platform classifications and are not necessarily mutually exclusive. Composite and heterojunction platforms may therefore contain materials listed in the preceding categories. Reported piezoelectric coefficients vary substantially with material composition, crystalline phase, molecular orientation, processing conditions, material dimensions, and measurement method and are therefore not directly compared. The listed biological functions have not necessarily been demonstrated simultaneously within every material system.

### 2.1 Inorganic piezoelectric materials

Among inorganic piezoelectric materials, piezoelectric ceramic barium titanate (BaTiO_3_, BTO), as a typical representative of lead-free perovskite piezoelectric materials, exhibits great potential in energy conversion and biomedical applications owing to its non-centrosymmetric tetragonal crystal structure and the pronounced polarization effect induced by the displacement of Ti^4+^ ions (Khare et al., 2020; Persano et al., 2024). Although its piezoelectric performance is currently inferior to that of lead-based materials, strategies such as elemental doping, grain-size regulation, and crystallographic orientation optimization can effectively enhance its properties. Meanwhile, BTO possesses excellent biocompatibility and nanoscale capability to traverse physiological barriers, enabling it to stand out in antibacterial therapy, tissue regeneration, and precision medicine applications. Zinc oxide (ZnO), a representative piezoelectric semiconductor, possesses a non-centrosymmetric hexagonal wurtzite structure with tetrahedral coordination. The alternating arrangement of Zn^2+^ and O^2-^ ions generates a piezoelectric potential under external mechanical stimulation. Although its bulk piezoelectric coefficient (d_33_) is only about 12 pm/V, ZnO has become a core material for piezoelectric nanogenerators (PENGs) and biomedical sensors owing to its excellent biocompatibility, low toxicity, and established safety in food and pharmaceutical applications (Ferrone et al., 2019; He and Briscoe, 2024; Wang Y. et al., 2022). In addition, molybdenum disulfide (MoS_2_), as a monolayer transition metal dichalcogenide, exhibits unique piezoelectric properties due to the non-centrosymmetric nature of its in-plane crystal structure (Hoàng Ly et al., 2025; Singh et al., 2019; Wang C. et al., 2022). Owing to its distinctive architecture composed of strong Mo–S covalent bonds and weak interlayer van der Waals interactions, MoS_2_ possesses a high specific surface area and excellent mechanical flexibility. Because molybdenum and sulfur elements exert beneficial effects on cellular functions, and the multivalent redox transitions of molybdenum confer strong catalytic activity, MoS_2_ demonstrates outstanding tunability and application potential in biomedicine, sensing, energy, and environmental science (Hoàng Ly et al., 2025; Singh et al., 2019; Wang C. et al., 2022).

### 2.2 Polymeric piezoelectric materials

Natural and polymeric macromolecules such as polyvinylidene fluoride (PVDF), poly-L-lactic acid (PLLA), and polyhydroxybutyrate (PHB) can also exhibit piezoelectricity when their molecular dipoles undergo reorientation under external stimulation. However, because the orientation of molecular dipoles in these polymers is less ordered than the crystal lattice of piezoelectric ceramics, their piezoelectric performance is generally inferior to that of piezoelectric ceramics (Khare et al., 2020; Zhang M. et al., 2023; Dai et al., 2022; Zaszczyńska et al., 2024; Goonoo et al., 2017; Zhang Y. Q. et al., 2023). Piezoelectric polymers generally offer favorable flexibility, processability, and biocompatibility; however, their biodegradability is highly material-dependent. PVDF and P(VDF-TrFE), for example, are generally non-biodegradable and may persist after implantation, whereas biodegradable piezoelectric polymers such as stereoregular polylactides and polyhydroxybutyrate (PHB) are more suitable for applications requiring temporary or resorbable electroactive scaffolds (Iwuozor et al., 2025; Zhang Z. et al., 2025). Polyvinylidene fluoride (PVDF) is one of the most extensively studied piezoelectric polymers. Owing to different arrangements of H and F atoms along the C–C backbone, PVDF can crystallize into multiple phases. Among them, the α phase possesses no net dipole moment and is therefore non-piezoelectric, whereas the β phase, characterized by an all-trans conformation with uniformly aligned dipoles, is the primary origin of its piezoelectricity (Pourmadadi et al., 2025; Mohammadpourfazeli et al., 2022). By regulating processing conditions or introducing nanofillers, the content of the β phase can be increased to enhance the piezoelectric performance. In particular, poly (vinylidene fluoride–trifluoroethylene) [P(VDF-TrFE)] (Pourmadadi et al., 2025; Mohammadpourfazeli et al., 2022) suppresses the formation of the α phase by introducing TrFE units that increase steric hindrance, thereby elevating the piezoelectric coefficient to approximately 30 pC N^−1^. Owing to its superior flexibility, remnant polarization, and electromechanical coupling properties, P(VDF-TrFE) shows distinct advantages in applications such as tissue engineering scaffolds and flexible biosensors. Because lactic acid is chiral, stereoregular polylactides, including poly-L-lactic acid (PLLA) and its enantiomer poly-D-lactic acid (PDLA), can exhibit shear piezoelectricity when their molecular chains are sufficiently oriented (Lotz et al., 2018; Yoshida et al., 2014). Their piezoelectric response originates from the non-centrosymmetric organization of chiral polymer chains and the orientation of carbonyl dipoles along the polymer backbone. Among these materials, PLLA has been particularly extensively investigated for biomedical and bone-tissue-engineering applications because of its biodegradability, biocompatibility, established processing methods, and ability to provide mechanically induced electrical cues. Unlike ferroelectric polymers such as PVDF, PLLA predominantly exhibits shear piezoelectricity, commonly described by the coefficient d_14_, and its magnitude is strongly influenced by molecular orientation, crystallinity, and processing conditions (Zaszczyńska et al., 2024; Mirghaffari et al., 2025; Schönlein et al., 2026; Zhang S. et al., 2024). Combined with its excellent biocompatibility, PLLA holds significant application potential in the biomedical field, particularly in bone tissue engineering, as its piezoelectric behavior closely matches the bioelectrical microenvironment generated by the oriented collagen fibers in bone tissue. Polyhydroxybutyrate (PHB) is another biodegradable and biocompatible piezoelectric polymer. Its non-centrosymmetric helical molecular organization gives rise to shear piezoelectricity, although its electromechanical properties depend strongly on crystallinity, molecular orientation, and processing conditions. These characteristics have supported the investigation of PHB and PHB-based composites as resorbable electroactive scaffolds for tissue-engineering applications (Zhang Z. et al., 2025).

### 2.3 Composite and heterojunction piezoelectric platforms

To overcome the intrinsic brittleness, limited deformability, or restricted functionality of individual piezoelectric materials, increasing attention has been directed toward composite and heterojunction platforms. Piezoelectric ceramics or semiconductors can be incorporated into polymer matrices, deposited onto implant surfaces, or integrated into porous scaffolds to combine electrical activity with improved mechanical flexibility, processability, and tissue compatibility. Ceramic–polymer composites, for example, can exploit the relatively strong piezoelectric response of inorganic phases while retaining the structural adaptability of polymeric matrices.

Piezoelectric heterojunctions provide an additional strategy for improving therapeutic performance. Through interfacial electric fields, band alignment, defect engineering, and enhanced separation and transfer of mechanically generated charge carriers, heterojunction structures can increase piezocatalytic activity and ROS generation. More complex architectures, including multifunctional scaffolds, Janus membranes, and hydrogels, further enable the integration of antibacterial, antibiofilm, immunomodulatory, drug-delivery, antioxidant, angiogenic, and osteogenic functions within a single platform. These systems are therefore particularly relevant to bone infection, in which bacterial eradication and tissue regeneration must often be addressed simultaneously or sequentially. However, increasing structural complexity also introduces challenges related to reproducibility, sterilization stability, degradation behavior, dose control, scale-up, and attribution of the biological effects to individual components.

A comparative overview of representative piezoelectric material platforms, their functional characteristics, advantages, limitations, and potential roles in bone infection therapy is provided in Table 1.

## 3 Mechanisms of the antibacterial activity of piezoelectric materials

Under mechanical stimulation, piezoelectric materials undergo deformation and polarization, resulting in charge separation and localized electrical potentials. In semiconducting or catalytically active piezoelectric systems, mechanically generated charge carriers can participate in surface redox reactions and promote the formation of reactive oxygen species (ROS), including hydroxyl radicals (•OH), superoxide radicals (•O_2_^−^), and, in selected systems, singlet oxygen (^1^O_2_) (Chen et al., 2023; Chen et al., 2026; Zhang X. et al., 2024). These electrical and catalytic effects can contribute to bacterial killing through multiple interconnected pathways, including disruption of biofilm architecture, damage to bacterial envelopes, oxidative injury to intracellular macromolecules, and interference with respiratory and energy-metabolic processes. Importantly, ROS generation is not universal to all piezoelectric materials and should not be regarded as the sole antibacterial mechanism. Depending on material composition and therapeutic design, electrical perturbation, mechanical disruption, interfacial electron transfer, ion release, cavitation, photothermal effects, and triggered drug delivery may also contribute to the overall antibacterial outcome (Huang et al., 2024; Wang et al., 2025; Feng et al., 2022; Yu et al., 2023; Chen C. et al., 2025). The major mechanically activated antibacterial mechanisms of piezoelectric materials are schematically summarized in Figure 1.

**FIGURE 1:**
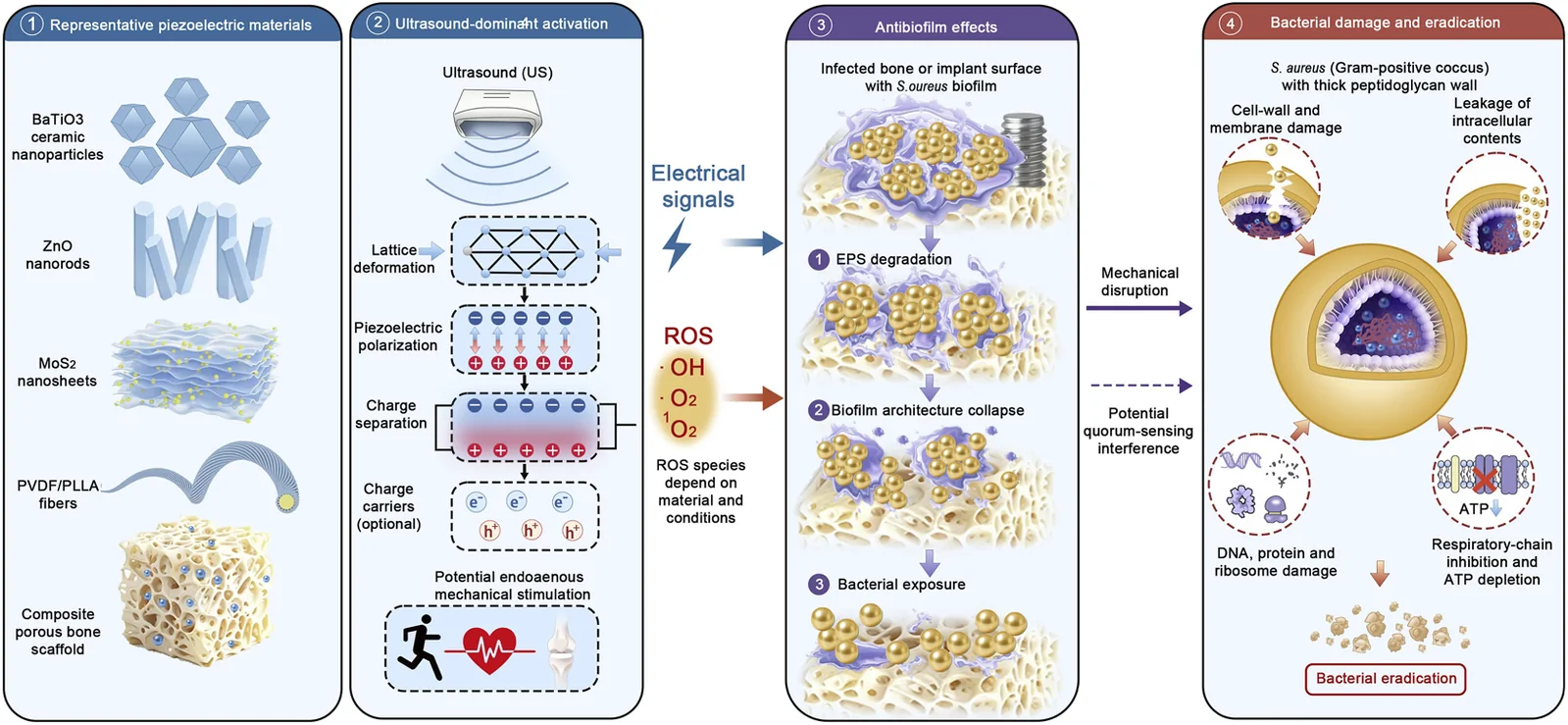
Ultrasound-dominant piezoelectric activation mediates biofilm disruption and bacterial eradication in bone infection. Representative piezoelectric materials, including BaTiO_3_ ceramic nanoparticles, ZnO nanorods, MoS_2_ nanosheets, PVDF/PLLA fibers, and composite porous bone scaffolds, undergo lattice deformation and piezoelectric polarization under ultrasound (US) or endogenous mechanical stimulation. The resulting charge separation generates localized electrical signals and, depending on material composition and reaction conditions, reactive oxygen species (ROS), including hydroxyl radicals (•OH), superoxide radicals (•O_2_^−^), and singlet oxygen (^1^O_2_). These effects promote extracellular polymeric substance (EPS) degradation, biofilm-architecture collapse, and exposure of previously protected bacteria. Subsequent mechanical disruption, electrical perturbation, and ROS-mediated oxidative damage compromise bacterial cell-wall and membrane integrity, damage DNA, proteins, and ribosomes, inhibit the respiratory chain, deplete ATP, and ultimately eradicate bacteria. *Staphylococcus aureus*, *Staphylococcus aureus*; ROS, reactive oxygen species; EPS, extracellular polymeric substances; PVDF, poly (vinylidene fluoride); PLLA, poly (L-lactic acid).

At present, the antibacterial mechanisms of piezoelectric materials mainly involve damage to biofilms, bacterial membranes, bacterial cell walls, and intracellular DNA or proteins. Bacterial biofilms are highly organized three-dimensional communities composed of microbial cells and their secreted extracellular polymeric substances, representing an important strategy for bacterial survival and persistent infection under adverse conditions (Chen et al., 2023). Biofilm formation is generally a dynamic, reversible, and multistage process, which can be summarized into four key stages (Costerton et al., 1999): (1) initial attachment; (2) irreversible attachment and microcolony formation; (3) biofilm maturation; and (4) detachment and dispersion.

### 3.1 Biofilm disruption

Bacterial biofilms represent a major barrier to successful treatment of bone infection because extracellular polymeric substances (EPS) restrict antimicrobial penetration and protect embedded bacteria from both host immune responses and antimicrobial agents (Masters et al., 2019; Costerton et al., 1999). Mechanically activated piezoelectric systems can interfere with biofilms through several complementary mechanisms. First, ROS generated during piezocatalysis can oxidatively damage EPS components and bacterial cells, thereby weakening the structural integrity of the biofilm and increasing bacterial exposure (Chen et al., 2023; Chen et al., 2026; Zhang X. et al., 2024). Second, ultrasound-associated mechanical effects, including acoustic forces and, in some systems, cavitation or material propulsion, can promote physical disruption of biofilm architecture and improve contact between antibacterial materials and bacteria (Feng et al., 2022; Chen C. et al., 2025). In addition, mechanically generated electrical signals and interfacial charge transfer may interfere with bacterial adhesion, membrane electrochemical homeostasis, and biofilm-associated metabolic processes (Zhang X. et al., 2024; Yu et al., 2023).

These mechanisms are highly material- and stimulation-dependent. For example, the MoS_2_-containing system reported by Feng et al. combined piezoelectric charge separation with ultrasound-driven propulsion, whereas the microbubble–piezoelectric scaffold developed by Chen et al. relied substantially on ultrasound-induced cavitation and enhanced drug penetration (Feng et al., 2022; Chen C. et al., 2025). Therefore, biofilm disruption observed in multifunctional piezoelectric systems should not automatically be attributed solely to intrinsic piezoelectricity.

### 3.2 Damage to bacterial membranes and cell envelopes

The bacterial envelope represents another major target of piezoelectric antibacterial systems. ROS generated by piezocatalytic reactions can oxidize membrane lipids and proteins, resulting in increased membrane permeability, loss of membrane potential, leakage of intracellular constituents, and ultimately loss of bacterial viability (Chen et al., 2023; Zhang X. et al., 2024; Huang et al., 2024; Wang et al., 2025). Huang et al., for example, demonstrated that piezoelectrically enhanced ROS production caused bacterial membrane damage and facilitated Cu influx, whereas Wang et al. reported membrane rupture and protein leakage in a defect-engineered piezoelectric heterojunction system (Huang et al., 2024; Wang et al., 2025).

In addition to oxidative damage, localized electrical fields and mechanically induced interfacial interactions may disturb membrane electrochemical homeostasis and increase envelope permeability. In some multifunctional platforms, metal-ion release further contributes to membrane and protein damage. These mechanisms frequently act in parallel, making it difficult to assign the observed antibacterial effect to a single pathway. Therefore, mechanistic interpretation should distinguish intrinsic piezoelectric effects from the contributions of ROS-producing catalytic components, released ions, and other incorporated antibacterial agents.

### 3.3 Intracellular macromolecular damage and metabolic interference

Following disruption of the bacterial envelope, oxidative stress and charge-transfer processes can further affect intracellular macromolecules and energy metabolism. Excessive ROS can induce oxidative damage to DNA, proteins, ribosomal components, and membrane-associated respiratory enzymes, thereby interfering with bacterial replication, protein synthesis, and metabolic homeostasis (Chen et al., 2023; Zhang X. et al., 2024; Wang et al., 2025; Yu et al., 2023). In the PCN-222/BTO heterojunction reported by Yu et al., ultrasound-induced interfacial electron transfer not only enhanced ROS generation but also promoted electron extraction from the bacterial respiratory chain, accompanied by damage to DNA and ribosomes and suppression of ATP production (Yu et al., 2023). Similarly, Wang et al. observed respiratory-chain disruption and glutathione depletion in a defect-engineered TiO_2_/BaTiO_3_ heterojunction system (Wang et al., 2025).

Other multifunctional platforms can interfere with bacterial metabolism through additional pathways. For example, Huang et al. reported that piezoelectrically enhanced Cu redox cycling promoted membrane damage and Cu influx, leading to disruption of the tricarboxylic acid cycle and a cuproptosis-like antibacterial response (Huang et al., 2024). These findings illustrate that inhibition of bacterial energy metabolism may arise from a combination of oxidative damage, electron-transfer disturbance, metal-ion-associated toxicity, and loss of membrane electrochemical integrity rather than from piezoelectricity alone.

### 3.4 Mechanistic integration and limitations

Collectively, piezoelectric antibacterial activity should be interpreted as a platform-dependent combination of electrical, catalytic, mechanical, and biochemical effects rather than as a single ROS-mediated process. In relatively simple piezoelectric systems, mechanical polarization and charge separation may represent the dominant initiating events. In contrast, many recently developed biomedical platforms incorporate heterojunctions, metal ions, sonosensitizers, nanozymes, photothermal components, antibiotics, or other functional agents, making the resulting antibacterial effects inherently multimodal (Huang et al., 2024; Wang et al., 2025; Feng et al., 2022; Yu et al., 2023; Chen C. et al., 2025; Zhang G. et al., 2025; Roy et al., 2025; Xi et al., 2025; Pan et al., 2026). Although such integration can substantially enhance therapeutic efficacy, it also complicates mechanistic attribution. Appropriate control groups that separate ultrasound, piezoelectric polarization, ROS production, ion release, thermal effects, and drug delivery are therefore important for determining the specific contribution of piezoelectricity in future studies.

From a translational perspective, this mechanistic complexity also highlights the need to balance antibacterial potency with host-cell safety. Strong ROS generation may facilitate rapid bacterial eradication, but excessive oxidative stress can also damage immune and osteogenic cells. Conversely, insufficient piezoelectric or catalytic activation may fail to disrupt mature biofilms. Thus, the therapeutic value of piezoelectric materials depends not only on maximizing antibacterial output but also on controlling its magnitude, duration, and timing within an appropriate therapeutic window.

To place these mechanisms in the broader context of antibacterial biomaterials for bone infection, piezoelectric strategies should be compared with established and emerging approaches rather than considered in isolation. Local antibiotic-delivery systems remain clinically relevant because they can provide high antimicrobial concentrations at the infection site, whereas metal-ion-based materials, photothermal therapy, photodynamic therapy, sonodynamic therapy, and chemodynamic or nanozyme-based systems offer different antibiotic-independent mechanisms (Zhang H. et al., 2025). Each strategy has distinct advantages and limitations with respect to tissue penetration, controllability, antimicrobial spectrum, cytotoxicity, and compatibility with bone regeneration (Kong et al., 2025). A comparison of representative strategies is summarized in Table 2.

**TABLE 2:** Representative piezoelectric platforms evaluated for antibacterial, immunomodulatory, and regenerative applications relevant to bone infection.

Study	Piezoelectric system/form	Activation	Disease or model context	Antibacterial/antibiofilm mechanism	Immunomodulatory mechanism	Regenerative outcome	Key limitations/considerations
(Wu et al., 2024)	Curcumin-loaded, Fe-doped BTO nanoparticles coated with γ3-peptide-engineered mesenchymal-stem-cell membranes (BFTO-Cur@EMM), incorporated into a 3D-printed quaternized-chitosan/tricalcium-phosphate scaffold	Alternating magnetic field for magnetic activation; LIPUS for piezoelectric stimulation	Icam1^+^ macrophage-associated infectious bone defects	Magnetic actuation facilitates bacterial-biofilm disruption and enhances local antibacterial treatment	Restores OXPHOS in Icam1^+^ macrophages through JAK2–STAT3 activation and MAPK–JNK suppression, promoting pro-reparative functional remodeling	Enhances secretion of angiogenic and osteogenic factors and promotes infected bone-defect regeneration	Targeted membrane-coated multimodal system involves complex fabrication; reproducibility, sterilization, and large-scale manufacture require evaluation
(Huang et al., 2024)	Cu-loaded PDA-coated morphotropic Ba_0_._90_Ca_0_._10_Ti_0_._91_Zr_0_._09_O_3_ piezo-hot nanoreactors integrated into an HA-modified 3D-printed PEKK scaffold	Ultrasound	Implant-associated bacterial infection	Piezoelectric charge generation and electron transfer promote Cu^2+^/Cu^+^ cycling and amplify ROS production; membrane damage and Cu influx disrupt the bacterial TCA cycle and induce cuproptosis-like bacterial death	Not investigated	Ca-, P-, and Cu-associated signaling supports angiogenesis, osteogenic differentiation, and infected implant–bone integration	The respective antibacterial and regenerative contributions of the piezoelectric material, Cu redox cycle, and scaffold matrix require further separation
(Zheng et al., 2025)	PLGA/Zn-KNN hybrid piezoelectric scaffold	Ultrasound	Bone-infection model	Ultrasound-induced electrical stimulation combined with Zn^2+^ release produces direct bacterial killing	Promotes macrophage M1 polarization, phagocytosis, and antibacterial activity; transcriptomic changes involve complement/coagulation and cytokine-related pathways	Improves the local bone microenvironment and promotes new-bone formation	M1-associated activation may aid early infection control but be detrimental if prolonged; temporal regulation requires clarification
(Xu et al., 2025)	Phase- and defect-engineered tetragonal barium strontium titanate containing oxygen vacancies (T-BSTO-VO)	Intensity-dependent ultrasound	MRSA-infected bone defects	High-intensity ultrasound enhances charge separation and •OH-dominant piezo-sonocatalysis, producing efficient MRSA eradication	Reduced infection-associated inflammation is observed, but no direct macrophage target or immune pathway is established	Mild ultrasound promotes Schwann-cell neural differentiation and neurotrophic signaling; increased intracellular Ca^2+^ and PI3K–AKT-associated neuro-osteogenic coupling enhance BMSC osteogenesis	Antibacterial and regenerative effects require different ultrasound intensities; Ca^2+^–PI3K–AKT represents a neuro-osteogenic rather than macrophage pathway
(Fan et al., 2025)	TiO_2_/Bi_2_WO_6_ piezoelectric heterojunction array constructed on a titanium implant	808-nm near-infrared irradiation for antibacterial therapy; endogenous cell-generated mechanical forces for piezoelectric osteogenic stimulation	Antibacterial implant and bone–implant osseointegration models	NIR-triggered photodynamic and photothermal effects produce ROS and heat for bacterial elimination	Not investigated	Cell-generated mechanical forces activate piezoelectric electrical signals, promote Ca^2+^ influx and PI3K–AKT signaling, and enhance osteogenic differentiation and osseointegration	Antibacterial activity is primarily NIR-driven, whereas regenerative stimulation is mechanically activated; the two activation modalities should not be conflated
(Cheng et al., 2025)	Double-layered asymmetric Janus GBR membrane comprising a nonpiezoelectric PLLA gingival-facing layer and a piezoelectric PLLA–ZnO bone-facing layer	Ultrasound	*P. gingivalis*-associated periodontitis and alveolar-bone regeneration	Piezoelectric stimulation, ROS production, Zn^2+^ release, and physical bacterial isolation inhibit bacterial growth and biofilm formation	Not investigated	Supports cell adhesion and increases BMP2, RUNX2, OPN, and OCN expression, promoting alveolar-bone regeneration	Periodontitis and alveolar-bone evidence should not be directly generalized to long-bone osteomyelitis
(Wang et al., 2025)	Oxygen-vacancy- and Ti^3+^-rich deficient TiO_2_/BaTiO_3_ heterojunction coating fabricated on titanium	Ultrasound	Implant-associated bacterial infection and bone-regeneration model	Defect-induced spin polarization and heterojunction formation accelerate sono-piezoelectric charge transport and increase ^1^O_2_ and •OH production, causing membrane rupture, protein leakage, respiratory-chain disruption, GSH depletion, and biofilm eradication	Reduced inflammatory infiltration and a later increase in CD206^+^ M2-associated macrophages accompany infection control; no causal immune pathway is established	Ultrasound-induced microcurrents increase intracellular Ca^2+^, promote mitochondrial fusion, and enhance osteogenic differentiation and new-bone formation	Macrophage changes may partly reflect bacterial clearance rather than direct immune regulation; long-term fate of the defect-engineered coating requires investigation
(Zhang G. et al., 2025)	Berberine-integrated Cu-doped SrTiO_3_ piezoelectric coating (STO-Cu@Ber) on titanium	Ultrasound	Implant-associated osteomyelitis	Enhanced charge separation, ROS generation, and Cu redox cycling disrupt bacterial metabolism and induce cuproptosis-like bacterial death and biofilm eradication	Sequential immune response: early enhancement of M1-associated phagocytosis and antibacterial activity, followed by M2-associated repair and inflammatory resolution after bacterial clearance	Enhances angiogenic and osteogenic signaling and improves bone regeneration and osseointegration	Contributions of berberine, Cu doping, piezoelectric stimulation, and surface nanostructure require further separation; herbal-component standardization is needed
(Feng et al., 2022)	RBC-membrane-coated porphyrin-based hollow MOF sonosensitizer coupled with piezoelectric MoS_2_ (RBC-HNTM-MoS_2_)	Ultrasound	MRSA osteomyelitis	MoS_2_ polarization improves interfacial charge separation and ^1^O_2_/•O_2_^−^ generation; ultrasound-driven propulsion increases bacterial contact and mechanical disruption, while the RBC membrane neutralizes bacterial toxins	Reduces inflammatory infiltration and suppresses infection-associated inflammation; no specific immune-cell pathway was established	Reduces infection-associated bone destruction and promotes bone-tissue recovery	Multicomponent formulation, membrane-coating stability, and ultrasound-delivery parameters may complicate clinical translation
(Yu et al., 2023)	PCN-222/BTO piezoelectric heterojunction	Ultrasound	MRSA-infected osteomyelitis model	Abiotic and biotic interfacial electron transfer increases ROS production and extracts electrons from the bacterial respiratory chain, causing membrane, DNA, and ribosomal damage and suppressing ATP synthesis	Not investigated	Reduces infection-associated bone destruction and supports bone-tissue recovery	Direct immunomodulatory and osteogenic mechanisms were not investigated; regenerative improvement may primarily result from effective infection control
(Chen K. et al., 2025)	3D-printed ZnO nanoparticle-modified BTO/HA piezoelectric ceramic composite scaffold	LIPUS	Infected bone defects	Ultrasound-triggered piezoelectric ROS generation combined with Zn^2+^-associated antibacterial activity promotes bacterial and biofilm eradication	No direct immunomodulatory mechanism was investigated	Promotes adhesion, proliferation, and osteogenic differentiation of osteogenic-related cells and supports infected bone-defect repair	Contributions of BTO piezoelectricity, ZnO/Zn^2+^ release, HA, and ultrasound stimulation should be distinguished
(Roy et al., 2025)	TLR-upregulated macrophage-membrane-cloaked Fe^2+^-doped BiOI nanosheets (MFBOI)	Ultrasound	MRSA-infected wounds and osteomyelitis	Defect engineering enhances piezoelectric charge separation and ROS generation, resulting in MRSA and biofilm elimination	Promotes macrophage reprogramming toward an M2-associated reparative phenotype, particularly in infected wounds	Piezoelectric electrical signals activate Ca^2+^ channels in mesenchymal stromal cells, enhancing osteogenic differentiation and bone repair	Wound-healing immunomodulation and osteomyelitis-related bone regeneration should be reported separately; complex membrane-coated formulation may affect reproducibility
(Chen C. et al., 2025)	Vancomycin-loaded lipid microbubbles combined with a BaTiO_3_-coated porous titanium scaffold	LIPUS	Infected bone defects	Ultrasound-induced cavitation disrupts biofilms, increases biofilm permeability, and triggers local vancomycin release; the piezoelectric scaffold provides additional electrical stimulation	LIPUS-driven piezoelectric signals promote M2-associated macrophage polarization and improve the local immune microenvironment, although a single causal pathway has not been established	M2-associated immune regulation and scaffold-mediated electrical stimulation enhance osteoblast activity and new-bone formation	The antibacterial contribution of vancomycin release and cavitation should be distinguished from the intrinsic effect of the piezoelectric scaffold
(Xi et al., 2025)	Bismuth-vacancy-rich Bi_2_S_3_ piezoelectric nanozyme incorporated into a phenylboronic-acid-modified sodium-alginate–arginine hydrogel (VBS-PSA)	High- *versus* low-power ultrasound	*In vitro* antibacterial/biofilm and osteoblast assays; bone-regeneration-oriented proof-of-concept study	High-power ultrasound enhances sonodynamic and peroxidase-like ROS generation for bacterial and biofilm elimination	Not investigated	Low-power ultrasound generates electrical cues, whereas catalase-like activity reduces excessive ROS and generates oxygen, promoting osteoblast proliferation and differentiation	An infected bone-defect model has not been established; therapeutic window and ultrasound parameters require standardized validation
(Pan et al., 2026)	NaNbOx@CeO_2_ piezoelectric heterojunction incorporated into PEKK, forming a PNC composite	Ultrasound	*In vitro* infection-mimicking acidic conditions and osteoblast evaluation	Heterojunction formation and oxygen vacancies enhance piezoelectric charge separation, sonodynamic activity, and CeO_2_-associated peroxidase-like catalysis under acidic conditions, increasing ROS-mediated bacterial and biofilm killing	Not investigated	Ultrasound-induced piezoelectric electrical signals promote osteoblast proliferation and osteogenic differentiation	Evidence remains predominantly at the material and cellular levels; therapeutic efficacy and safety in an infected bone-defect model require validation

Abbreviations: AMF, alternating magnetic field; BMSC, bone-marrow mesenchymal stromal cell; BTO, barium titanate; GBR, guided bone regeneration; GSH, glutathione; HA, hydroxyapatite; KNN, potassium sodium niobate; LIPUS, low-intensity pulsed ultrasound; MOF, metal–organic framework; MRSA, methicillin-resistant *Staphylococcus aureus*; NIR, near-infrared; OXPHOS, oxidative phosphorylation; PDA, polydopamine; PEKK, polyetherketoneketone; PLLA, poly-L-lactic acid; PLGA, poly (lactic-co-glycolic acid); RBC, red blood cell; ROS, reactive oxygen species; TCA, tricarboxylic acid; TLR, Toll-like receptor.

## 4 Applications of piezoelectric materials in bone infection

Bone infection remains difficult to treat because bacterial persistence, biofilm formation, immune dysregulation, and impaired bone regeneration occur simultaneously and interact with one another. Conventional treatment mainly relies on surgical debridement and systemic or local antibiotic administration; however, mature biofilms, antimicrobial resistance, incomplete drug penetration, and treatment-related toxicity may compromise therapeutic efficacy. Piezoelectric materials provide a distinct strategy by converting mechanical stimulation into localized electrical signals and, in catalytically active systems, promoting reactive oxygen species (ROS) generation. Recent studies further indicate that these platforms can integrate direct antibacterial effects with immune regulation and tissue regeneration. Rather than discussing individual studies sequentially, the available evidence can be organized according to four major therapeutic functions: direct piezocatalytic antibacterial and biofilm-disrupting effects, immunoregulatory and immunometabolic regulation, antibacterial–regenerative coupling, and multimodal or stage-adaptive therapeutic design.

### 4.1 Direct piezocatalytic antibacterial and biofilm-disrupting strategies

A major application of piezoelectric materials in bone infection is the direct elimination of bacteria and disruption of mature biofilms through mechanically activated catalytic and electrical effects. In many systems, ultrasound-induced piezoelectric polarization enhances charge separation and promotes ROS generation, thereby damaging bacterial membranes, intracellular macromolecules, respiratory pathways, and biofilm architecture. Importantly, these effects are frequently amplified through heterojunction construction, defect engineering, metal-ion cycling, or enhanced interfacial electron transfer.

Huang et al. developed a Cu-loaded piezoelectric nanoreactor in which ultrasound-induced charge generation promoted Cu^2+^/Cu^+^ redox cycling and amplified ROS production. The resulting membrane damage and Cu influx disrupted the bacterial tricarboxylic acid cycle and induced a cuproptosis-like antibacterial response, leading to effective control of implant-associated infection (Huang et al., 2024). Wang et al. similarly used defect-engineered TiO_2_/BaTiO_3_ heterojunctions to accelerate sono-piezoelectric charge transport and increase ^1^O_2_ and •OH generation, resulting in membrane rupture, protein leakage, respiratory-chain disruption, glutathione depletion, and biofilm eradication (Wang et al., 2025).

Other platforms enhance antibacterial efficacy through interfacial electron transfer or mechanical interactions in addition to ROS production. Feng et al. coupled piezoelectric MoS_2_ nanosheets with a porphyrin-based hollow MOF, in which piezoelectric polarization enhanced charge separation and ROS generation, while ultrasound-driven propulsion increased bacterial contact and mechanical disruption (Feng et al., 2022). Yu et al. demonstrated that a PCN-222/BTO heterojunction could promote both abiotic and biotic interfacial electron transfer, enabling ROS generation and direct electron extraction from the bacterial respiratory chain, thereby damaging bacterial membranes, DNA, and ribosomes and suppressing ATP synthesis (Yu et al., 2023). Similarly, ZnO-modified BTO/HA scaffolds combine ultrasound-triggered piezoelectric ROS generation with Zn^2+^-associated antibacterial activity to enhance bacterial and biofilm eradication (Chen K. et al., 2025).

Collectively, these studies demonstrate that direct antibacterial activity in piezoelectric systems is rarely attributable to a single mechanism. ROS generation, electron transfer, metal-ion release, mechanical disruption, and material-specific catalytic effects frequently operate simultaneously. Although this multimodal activity can improve bacterial eradication, it also complicates mechanistic attribution and emphasizes the need for carefully designed control groups to distinguish the intrinsic contribution of piezoelectric polarization from accompanying catalytic or chemical effects.

### 4.2 Immunoregulatory and immunometabolic strategies

Beyond direct bacterial killing, an emerging feature of piezoelectric materials is their ability to modulate immune-cell function within the infected bone microenvironment. This property is particularly relevant because effective treatment requires not only bacterial elimination but also appropriate temporal regulation of host immunity. Early antimicrobial immune activation may facilitate bacterial clearance, whereas prolonged pro-inflammatory activity can impair tissue repair and osteogenesis.

Wu et al. developed a magneto-piezoelectric system targeting Icam1^+^ macrophages and demonstrated that low-intensity pulsed ultrasound restored macrophage oxidative phosphorylation through JAK2–STAT3 activation and MAPK–JNK suppression, promoting a pro-reparative functional phenotype and enhancing the secretion of angiogenic and osteogenic factors (Wu et al., 2024). In contrast, Zheng et al. showed that an ultrasound-driven PLGA/Zn-KNN scaffold enhanced M1-associated macrophage polarization and phagocytic activity during infection control, accompanied by activation of complement, coagulation, inflammatory, and cytokine-related pathways (Zheng et al., 2025). These findings illustrate that macrophage responses induced by piezoelectric stimulation may differ according to the biological stage and therapeutic objective.

Several multifunctional platforms further support this temporal concept. Zhang et al. reported a sequential immune response in which early enhancement of M1-associated phagocytosis and antibacterial activity was followed by M2-associated inflammatory resolution and tissue repair after bacterial clearance (Zhang G. et al., 2025). Roy et al. demonstrated macrophage reprogramming toward a reparative phenotype in a defect-engineered piezoelectric nanocomposite system (Roy et al., 2025), whereas Chen et al. reported that low-intensity pulsed ultrasound combined with a piezoelectric scaffold promoted M2-associated macrophage polarization and improved the local immune microenvironment (Chen C. et al., 2025).

These observations suggest that immunomodulation in bone infection should not be simplified to a preference for either “M1” or “M2” macrophages. Instead, the therapeutic requirement may change over time: antimicrobial macrophage activity may be beneficial during the early infection-control phase, whereas persistent inflammatory activation may become detrimental once bacterial burden has been reduced. Future piezoelectric systems should therefore aim to achieve temporally coordinated immune regulation rather than constitutive polarization toward a single macrophage phenotype.

### 4.3 Antibacterial–regenerative coupling

A distinctive advantage of piezoelectric biomaterials is their potential to connect infection control with tissue regeneration. Because bone itself is a mechanically responsive and bioelectrically active tissue, piezoelectric stimulation can provide electrical cues that influence osteogenic, angiogenic, and neurogenic processes after or alongside bacterial clearance.

Xu et al. developed an oxygen-vacancy-rich tetragonal barium strontium titanate system with intensity-dependent therapeutic functions. High-intensity ultrasound enhanced piezo-sonocatalytic ROS generation and MRSA eradication, whereas lower-intensity stimulation promoted Schwann-cell differentiation and Ca^2+^–PI3K–AKT-associated neuro-osteogenic signaling, thereby supporting BMSC osteogenesis (Xu et al., 2025). This work illustrates how stimulation intensity may be adjusted to shift the therapeutic emphasis from antibacterial activity toward regeneration.

Fan et al. constructed a TiO_2_/Bi_2_WO_6_ piezoelectric heterojunction implant in which near-infrared irradiation provided photodynamic and photothermal antibacterial effects, while mechanical forces generated by adherent cells activated local piezoelectric fields that promoted osteogenic differentiation and osseointegration (Fan et al., 2025). Cheng et al. used an asymmetric Janus membrane to achieve spatial functional separation, with one interface supporting antibacterial activity and the other promoting cell adhesion, osteogenic differentiation, and alveolar-bone regeneration (Cheng et al., 2025).

Intensity-dependent and microenvironment-responsive coupling has also been explored in multifunctional nanozyme systems. Xi et al. developed a bismuth-vacancy-rich Bi_2_S_3_ piezoelectric nanozyme hydrogel in which high-power ultrasound promoted ROS-mediated bacterial killing, whereas low-power ultrasound provided electrical stimulation together with catalase-like antioxidant activity to reduce excessive ROS and support osteoblast differentiation (Xi et al., 2025). Pan et al. similarly combined a NaNbO_x_@CeO_2_ piezoelectric heterojunction with PEKK, enabling ROS-mediated antibacterial activity together with Ce^3+^/Ce^4+^-associated antioxidant regulation and osteogenic stimulation (Pan et al., 2026).

Together, these studies indicate that successful antibacterial–regenerative coupling requires more than simultaneous enhancement of antibacterial and osteogenic effects. The intensity, timing, and microenvironmental context of stimulation are critical because strong oxidative activity that is beneficial for bacterial eradication may be detrimental to osteogenic and immune cells if maintained during the regenerative phase.

### 4.4 Multimodal and stage-adaptive therapeutic design

The increasing complexity of piezoelectric biomaterials reflects a broader transition from single-function antibacterial materials toward multimodal systems capable of addressing multiple pathological barriers in bone infection. Current platforms integrate piezoelectricity with magnetic actuation (Wu et al., 2024), metal-ion redox cycling (Huang et al., 2024; Zhang G. et al., 2025), photothermal and photodynamic therapy (Fan et al., 2025), nanozyme activity (Xi et al., 2025; Pan et al., 2026), toxin neutralization (Feng et al., 2022), or ultrasound-triggered drug delivery (Chen C. et al., 2025). Such combinations can improve antibacterial potency and extend therapeutic functionality, but they also increase fabrication complexity and make it more difficult to determine the specific contribution of each component.

More importantly, the available evidence supports a stage-adaptive therapeutic framework for piezoelectric treatment of bone infection. During the early infection-control phase, mechanically activated piezoelectric platforms should prioritize sufficient antibacterial output to disrupt biofilms, reduce bacterial burden, and support antimicrobial immune functions. Once infection is controlled, continued excessive ROS generation or inflammatory activation may become counterproductive. The therapeutic priority should then shift toward limiting oxidative injury, restoring cellular metabolism, resolving persistent inflammation, promoting pro-reparative immune functions, and supporting angiogenesis and osteogenesis.

Accordingly, the most promising future direction may not simply be to maximize the number of functions integrated into a single material, but to develop systems capable of dynamically changing their biological output according to the stage of treatment. Intensity-dependent activation, sequential release, microenvironment-responsive catalysis, and spatially compartmentalized designs may provide practical strategies for achieving such temporal control. Representative piezoelectric platforms and their antibacterial, immunomodulatory, regenerative, and mechanistic characteristics are summarized in Table 3.

**TABLE 3:** Comparison of representative antibacterial biomaterial strategies relevant to bone infection.

Antibacterial strategy	Major antibacterial mechanism	Principal advantages	Major limitations	Comparison with piezoelectric strategies
Local antibiotic-delivery biomaterials	Sustained or controlled local release of antibiotics; inhibition of bacterial growth and biofilm-associated bacteria	Clinically established antimicrobial agents; high local drug concentrations; reduced systemic exposure	Antibiotic resistance; drug-specific spectrum; burst or insufficient release; limited activity against mature biofilms; release kinetics depend on carrier	Greater clinical maturity than piezoelectric systems, but lacks intrinsic bioelectrical stimulation and remains dependent on antibiotics
Metal-ion-based biomaterials	Release of Ag^+^, Cu^2+^, Zn^2+^ or other ions causing membrane damage, protein dysfunction, oxidative stress, and metabolic disturbance	Broad-spectrum activity; relatively simple incorporation into scaffolds; some ions also promote osteogenesis or angiogenesis	Narrow therapeutic window; dose-dependent cytotoxicity; potential accumulation; difficult long-term control of ion release	Frequently combined with piezoelectric materials; ion effects must be distinguished from intrinsic piezoelectric antibacterial effects
Photothermal therapy (PTT)	Light-to-heat conversion causes membrane/protein damage and bacterial death	Rapid and broad-spectrum killing; low susceptibility to conventional antibiotic resistance	Limited light penetration into deep bone; risk of thermal injury; requires control of local temperature	Piezoelectric systems can be activated by ultrasound or mechanical forces with greater applicability to deep tissues and generally avoid reliance on sustained high temperature
Photodynamic therapy (PDT)	Light-activated photosensitizers generate ROS	Non-antibiotic mechanism; spatially controllable; low tendency for conventional resistance	Limited light penetration; dependence on photosensitizer localization and local oxygen; potential phototoxicity	Both can rely on ROS, but piezocatalysis uses mechanical rather than optical activation and may additionally provide electrical stimulation
Sonodynamic therapy (SDT)	Ultrasound activates sonosensitizers to generate ROS and cavitation-associated antibacterial effects	Deeper tissue penetration than light-based approaches; noninvasive external activation	Depends on sonosensitizer efficiency, ultrasound parameters, oxygen availability, and cavitation; mechanism can be heterogeneous	Shares ultrasound activation with many piezoelectric systems; piezoelectric materials additionally convert mechanical energy into electrical polarization, although piezoelectric and sonodynamic contributions may overlap
Chemodynamic/nanozyme-based therapy	Catalytic conversion of endogenous substrates into toxic ROS, often through Fenton or Fenton-like reactions	Can exploit the infection microenvironment; no external light source necessarily required	Strong dependence on local pH, H_2_O_2_ concentration, catalytic metal availability, and host-cell tolerance	Can be integrated with piezoelectric systems to amplify ROS, but increases mechanistic and compositional complexity
Piezoelectric/piezocatalytic therapy	Mechanical-energy conversion into electrical polarization, charge separation, localized electrical cues, and, in catalytically active systems, ROS; may also promote interfacial electron transfer and mechanical disruption	Remote mechanical activation; potential deep-tissue stimulation; antibiotic-free operation; ability to couple antibacterial activity with immunomodulatory and regenerative bioelectrical cues	Piezoelectric output and ROS generation vary markedly among materials; stimulation parameters lack standardization; excessive ROS may damage host cells; contribution of piezoelectricity can be difficult to separate in multifunctional platforms; limited clinical evidence	Distinctive potential lies in coupling mechanically triggered antibacterial activity with bioelectrical regulation of the infected bone microenvironment and regeneration

Based on this cross-study synthesis, we propose a stage-adaptive therapeutic framework for piezoelectric treatment of bone infection (Figure 2). During the early infection-control phase, mechanically activated piezoelectric platforms should provide sufficient electrical cues and controlled ROS generation to disrupt biofilms, eradicate bacteria, and enhance antimicrobial macrophage functions. Following bacterial clearance, the therapeutic priority should shift toward limiting excessive oxidative injury, restoring cellular metabolism, resolving persistent inflammation, and promoting pro-reparative macrophage functions. This restored microenvironment may subsequently support angiogenesis, mesenchymal stromal-cell osteogenic differentiation, bone remodeling, and vascularized bone regeneration. Importantly, this framework should be regarded as a conceptual design principle derived from cross-study evidence rather than as a therapeutic sequence that has already been demonstrated within every individual piezoelectric platform.

**FIGURE 2:**
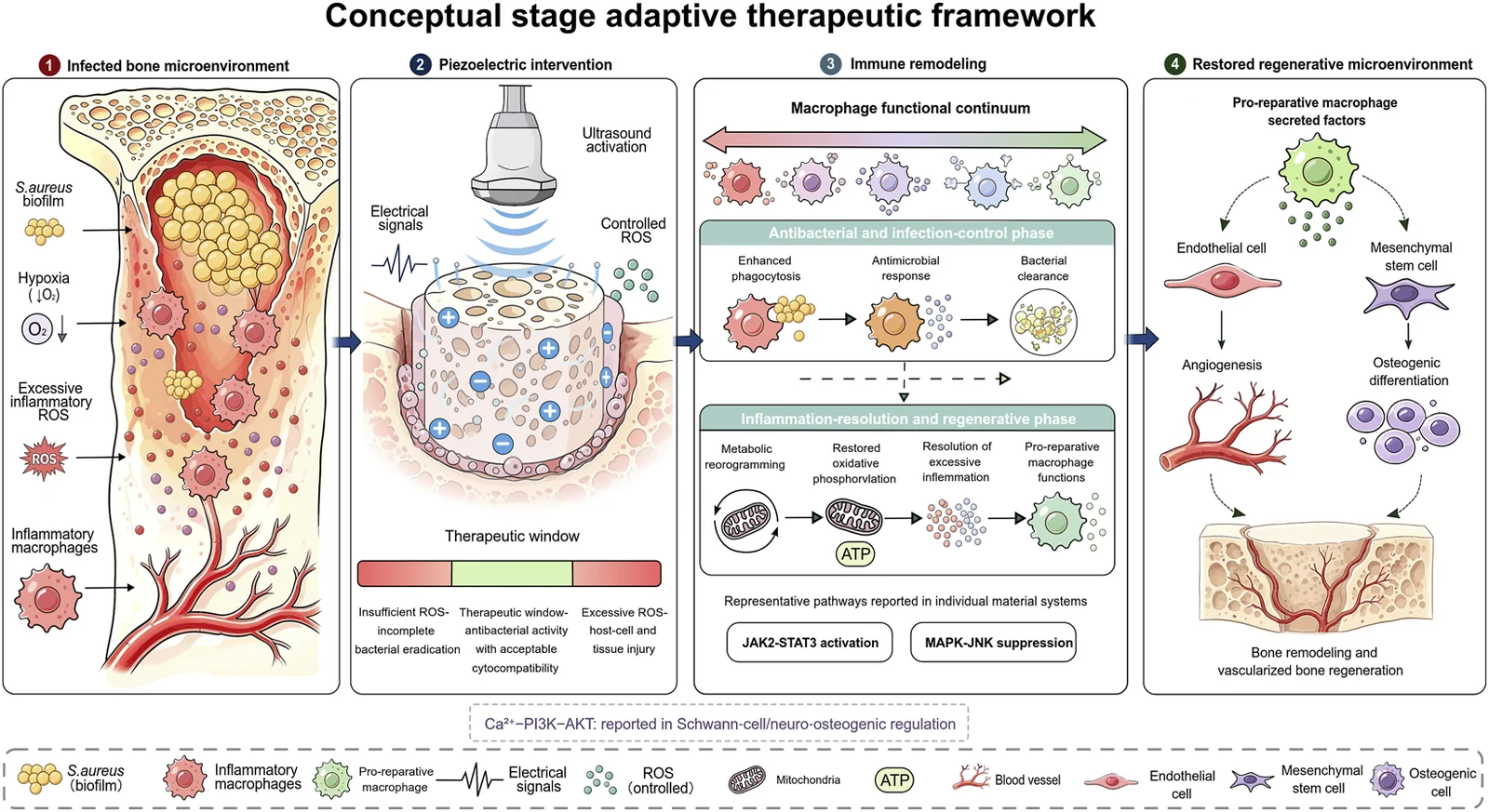
Conceptual stage-adaptive therapeutic framework for piezoelectric treatment of bone infection. Piezoelectric intervention, predominantly activated by ultrasound, converts mechanical energy into localized electrical signals and, in catalytically active systems, promotes controlled ROS generation within a defined therapeutic window. In the infected bone microenvironment, characterized by *Staphylococcus aureus* biofilm, hypoxia, excessive ROS, and inflammatory macrophages, the early antibacterial phase enhances macrophage phagocytosis and antimicrobial responses to facilitate bacterial clearance. Subsequently, restoration of oxidative phosphorylation and metabolic reprogramming promotes inflammation resolution and pro-reparative macrophage functions. Reported mechanisms in individual material systems include JAK2–STAT3 activation and MAPK–JNK suppression; Ca^2+^–PI3K–AKT signaling is associated with Schwann-cell-mediated neuro-osteogenic regulation. The restored microenvironment supports angiogenesis, mesenchymal stem-cell osteogenic differentiation, bone remodeling, and vascularized bone regeneration. ROS, reactive oxygen species; ATP, adenosine triphosphate.

## 5 Challenges and future perspectives

Despite the rapid development of piezoelectric materials for bone infection therapy, several key challenges must be addressed before clinical translation can be realized. First, the long-term biosafety of electrically active and ROS-generating materials remains insufficiently defined, particularly with respect to chronic exposure, material degradation, and potential accumulation *in vivo*. Second, antibacterial efficacy is highly dependent on the magnitude and duration of piezoelectric output and ROS generation, yet excessive ROS may compromise osteogenic and immune cells, whereas insufficient levels fail to eradicate bacteria. Achieving precise, adaptive regulation of piezoelectric activity therefore represents a central design objective. In parallel, the heterogeneous and dynamic nature of the infected bone microenvironment—characterized by biofilms, hypoxia, necrosis, and immune dysregulation—poses additional barriers that are not adequately captured by current experimental models. Beyond these practical limitations, a deeper mechanistic understanding of how piezoelectric-induced electrical cues coordinate antibacterial action, immune modulation, angiogenesis, and osteogenesis is still lacking. Clarifying these interconnections will be essential for transitioning from dual-function materials toward systems that actively orchestrate the entire healing cascade. From a translational standpoint, scalable fabrication, reproducibility, sterilization compatibility, and regulatory compliance remain critical considerations, particularly for complex multi-component architectures.

In the future, piezoelectric materials are expected to evolve into intelligent, self-powered therapeutic platforms capable of harvesting physiological mechanical energy to deliver on-demand antibacterial and regenerative cues. Integration with immunoengineering, metabolic modulation, and bioelectronic medicine may further establish a new paradigm for antibiotic-free management of refractory bone infections. With continued interdisciplinary efforts, piezoelectric-based strategies hold strong promise to become a cornerstone technology for next-generation infection-responsive biomaterials.

## 6 Conclusion

Bone infection remains a major clinical challenge due to biofilm formation, antibiotic resistance, and impaired bone regeneration. Emerging evidence demonstrates that piezoelectric materials offer a unique, non-antibiotic strategy by converting mechanical stimulation into localized electrical signals and, in catalytically active systems, promoting reactive oxygen species generation, enabling efficient bacterial eradication and biofilm disruption. Beyond direct antibacterial effects, emerging studies indicate that piezoelectric stimulation may also regulate infection-associated immune responses and support osteogenic and angiogenic repair. From the perspective proposed in this review, the therapeutic value of piezoelectric materials may therefore extend beyond the simultaneous integration of multiple functions toward the stage-dependent coordination of infection control and tissue regeneration. Future piezoelectric systems should evolve from static “antibacterial plus regenerative” designs toward stage-adaptive platforms that prioritize biofilm eradication and antimicrobial immune activity during early infection control, while subsequently promoting oxidative homeostasis, inflammation resolution, pro-reparative immune remodeling, angiogenesis, and osteogenesis. This pathophysiology-oriented framework may provide a useful design principle for next-generation piezoelectric biomaterials for refractory bone infections.
